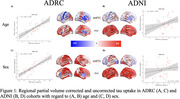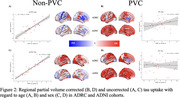# A multiple cohort study exploring the influence of partial volume correction on age and sex effects on tau PET imaging

**DOI:** 10.1002/alz.093175

**Published:** 2025-01-09

**Authors:** Diana A Hobbs, Shaney Flores, Sarah J. Keefe, John C. Morris, Tammie L.S. Benzinger, Brian A. Gordon

**Affiliations:** ^1^ Washington University in St. Louis, St. Louis, MO USA; ^2^ Washington University School of Medicine, St. Louis, MO USA

## Abstract

**Background:**

Tau PET imaging has become pivotal in understanding the pathophysiological processes underlying Alzheimer disease (AD). In individuals without amyloid pathology, there is evidence tau levels are elevated with increase age and that females show greater levels of binding. An unknown question is how consistent these effects are, and whether they are susceptible to methodological choices impacting PET quantification. We explored the impact of partial volume correction (PVC) on tau regional values in data from the Charles F. and Joanne Knight Alzheimer Disease Research Center (ADRC) and the Alzheimer Disease Neuroimaging Initiative (ADNI).

**Method:**

Data came from 323 participants (age: 67.89±8.57) at the Knight ADRC and 274 individuals (age: 70.14±6.61) from ADNI. Forty‐two regional values of tau PET binding were measured using flortaucipir with and without partial‐volume correction as implemented using study specific geometric transfer matrix approaches. Participants were selected to be amyloid‐negative. Similarity in regional estimates was determined using spatial permutation testing.

**Result:**

Within the ADRC cohort the value of the regression coefficients changed with PVC by the spatial pattern of the effects remained highly similar for both age (Figure 1A: r=0.66, p<0.001) and sex (Figure 1C: r=0.85, p<0.001). In ADNI there were moderate association between the maps of age‐related effects (Figure 1B: r=0.36, p<0.05) and sex (Figure 1D: r=0.31, p<0.05). When comparing between the ADRC and ADNI cohorts using non‐PVC data there were highly significant associations for both age (Figure 2A: r=0.83, p<0.001) and sex (Figure 2C: r=0.95, p<0.001). When using PVC, data no significant associations were found for age (Figure 2B: r=0.32, p=0.06) or sex (Figure 2D: r=0.02, p=0.92).

**Conclusion:**

The implementation of, and choice of PVC approach can drastically impact the detection of PET signals. This is a particular concern in the context of cohorts where there may be low to moderate levels of underlying pathology and where non‐specific binding may dominate the signal. These results emphasize the importance of considering PVC in tau PET analyses and highlight the reliability of observed age‐ and sex‐related effects across different cohorts.